# Pou4f1-Tbr1 transcriptional cascade controls the formation of Jam2-expressing retinal ganglion cells

**DOI:** 10.3389/fopht.2023.1175568

**Published:** 2023-05-18

**Authors:** Takae Kiyama, Halit Y. Altay, Tudor C. Badea, Chai-An Mao

**Affiliations:** ^1^ Ruiz Department of Ophthalmology and Visual Science, McGovern Medical School at The University of Texas Health Science Center at Houston (UTHealth), Houston, TX, United States; ^2^ Research and Development Institute, Transilvania University of Brasov, School of Medicine, Brasov, Romania; ^3^ National Center for Brain Research, Research Institute for Artificial Intelligence, Romanian Academy, Bucharest, Romania; ^4^ The MD Anderson Cancer Center/UTHealth Graduate School of Biomedical Sciences, Houston, TX, United States

**Keywords:** retinal ganglion cell, J-RGC, transcription regulation, CUT&Tag, Pou4f1, Tbr1, Jam2

## Abstract

More than 40 retinal ganglion cell (RGC) subtypes have been categorized in mouse based on their morphologies, functions, and molecular features. Among these diverse subtypes, orientation-selective Jam2-expressing RGCs (J-RGCs) has two unique morphologic characteristics: the ventral-facing dendritic arbor and the OFF-sublaminae stratified terminal dendrites in the inner plexiform layer. Previously, we have discovered that T-box transcription factor *T-brain 1* (*Tbr1*) is expressed in J-RGCs. We further found that *Tbr1* is essential for the expression of *Jam2*, and Tbr1 regulates the formation and the dendritic morphogenesis of J-RGCs. However, Tbr1 begins to express in terminally differentiated RGCs around perinatal stage, suggesting that it is unlikely involved in the initial fate determination for J-RGC and other upstream transcription factors must control *Tbr1* expression and J-RGC formation. Using the Cleavage Under Targets and Tagmentation technique, we discovered that Pou4f1 binds to *Tbr1 on* the evolutionary conserved exon 6 and an intergenic region downstream of the 3’UTR, and on a region flanking the promoter and the first exon of *Jam2*. We showed that Pou4f1 is required for the expression of *Tbr1 and Jam2*, indicating Pou4f1 as a direct upstream regulator of *Tbr1 and Jam2*. Most interestingly, the Pou4f1-bound element in exon 6 of *Tbr1* possesses high-level enhancer activity, capable of directing reporter gene expression in J-RGCs. Together, these data revealed a *Pou4f1-Tbr1-Jam2* genetic hierarchy as a critical pathway in the formation of J-RGC subtype.

## Introduction

Retinal ganglion cells (RGCs) are the output neurons of the retina that collectively transmit visual information to the brain. In a mature mouse retina, researchers have identified over 40 subtypes of RGCs, categorized by their unique morphology, function, and molecular profile. The discovery of these diverse RGC subtypes has led to intensive research using mouse retina as a model system to better understand the molecular and cellular mechanisms that govern the specification, maturation, and terminal differentiation of various neuronal subtypes in the central nervous system during development (1–13). Each of these RGC subtypes harbors a complex yet stereotypic dendritic morphology that synapses with bipolar and amacrine cells in precise laminar positions in the inner plexiform layer (IPL), and an axon that projects to multiple areas in the brain. RGCs function as an information processing hub and relay between the retina and the brain to transduce complex visual information (14, 15). Although a number of transcription factors (TFs) have been identified as key developmental regulators for initial RGC specification (16–20), little is known about the cellular and molecular mechanisms controlling the differentiation and maturation of RGC subtypes during development.

Previously, we and others have discovered that the expression of T-box transcription factor *Tbr1* marks 2 morphologically distinct groups of RGCs (symmetrical and asymmetrical), which share similar dendritic stratification positions in the IPL and project to the dorsal lateral geniculate nuclei and superior colliculus (5, 21). Through loss-of-function studies, we found that *Tbr1* is required for the expression of Jam2, and is essential for the formation for most of these RGC subtypes. The few surviving *Tbr1*-deleted RGCs develop abnormal and mis stratified dendrites. By gain-of-function studies, we found that ectopically expressing Tbr1 alone is sufficient to activate *Jam2 and* instruct M4-ipRGCs to alter their dendritic branching morphogenesis (5). While Tbr1’s function in the development and maturation of J-RGCs has been well studied, how J-RGCs arise from naïve RGCs remains illusive. Based on birth dating data (the time their progenitor cells exit cell cycle), Tbr1-expressing RGCs are born between E12 and E15, indicating that they are early born RGCs. However, lineage tracing using tamoxifen-treated *Tbr1^CreERT2^:Ai9* embryos at different time points showed that Tbr1-expressing cells in E14.5 developmental retinas do not give rise to Tbr1-expressing RGCs in mature retinas (5), suggesting the presence of other transcription factors upstream of Tbr1 responsible for fate determination of Tbr1-expressing J-RGCs.

**Figure 1:**
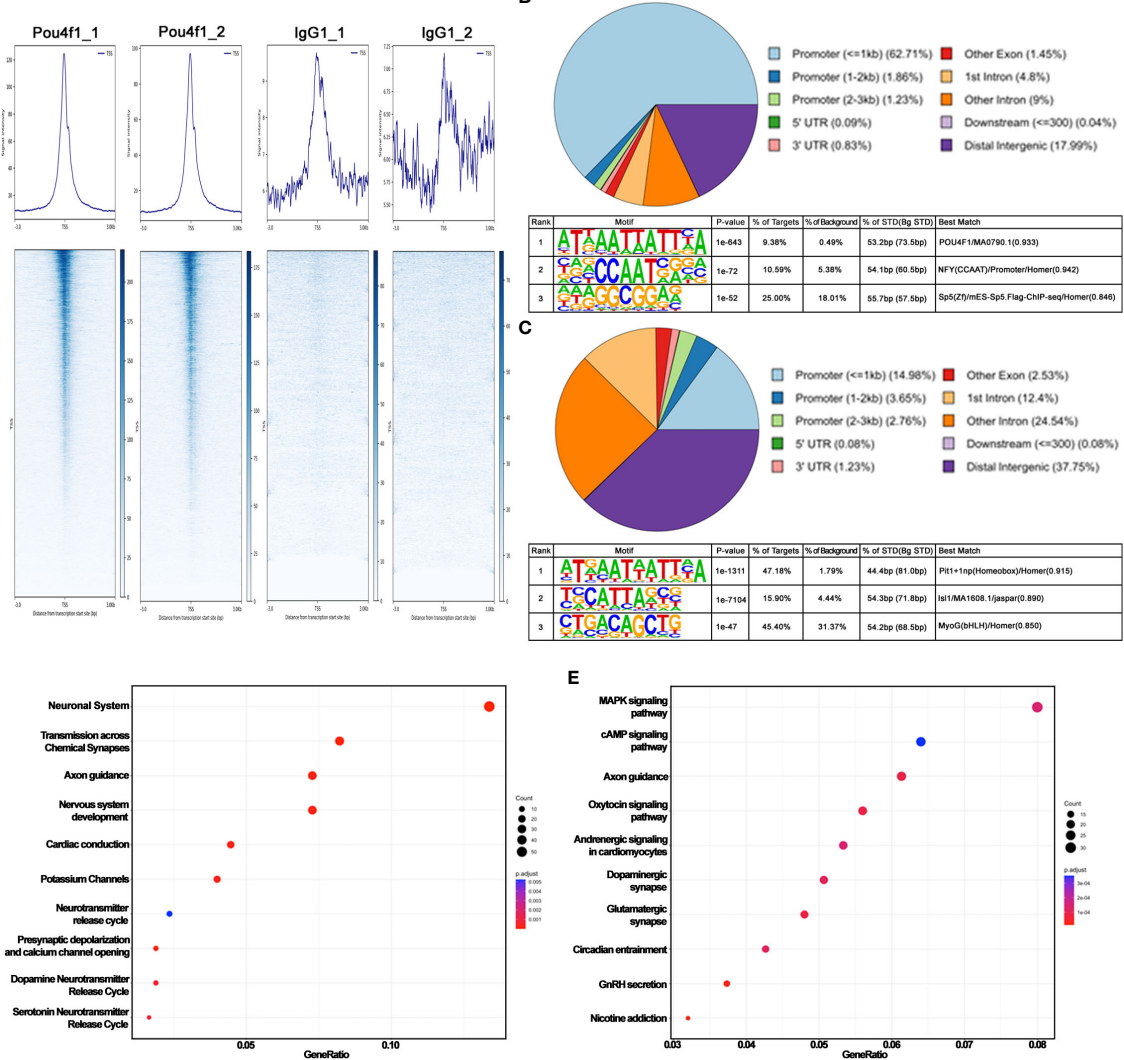
CUT&Tag sequencing reveals Pou4f1 occupancy in E16.5 RGCs. **(A)** Average density profiles (top) and heatmaps (bottom) showing Pou4f1 and IgG CUT&Tag signals in relation to transcription start site (TSS) ( ± 3 kb). **(B)** Motif enrichment analysis by HOMER of Pou4f1-bound elements displayed in **(A)**. **(C)** Motif enrichment analysis by HOMER of Pou4f1-bound elements in panel **(A)** intersected with RGC-specific open chromatin regions. **(D)** Reactome pathway analysis of genes associated with RGC-specific Pou4f1-bound elements in panel **(C)**. The top 10 most enriched reactome gene terms are presented using a dot-plot. **(E)** KEGG pathway analysis of genes associated with RGC-specific Pou4f1-bound elements in panel **(C)**. The top 10 most enriched KEGG terms are displayed using a dot-plot. X-axis indicates the gene ratio in the term, and the Y-axis indicates the category term. The size of the dots represents the number of genes found in each category term. The color of the dots represents the adjusted *P* value.

We hypothesized Pou4f1 is such an early TF because when *Jam2^CreERT2^* was genetically intersected with *Pou4f1^CKOAP/+^* at E14.5 retinas, J-RGC was the predominant RGC subtype that appeared in mature retina, and Tbr1-expressing RGCs are expressed exclusively in Pou4f1-expressing RGCs in adult retinas (5). These observations suggested a lineal relationship between Pou4f1 and Tbr1. Pou4f1, a class IV POU domain-containing transcription factor, is expressed in differentiated RGCs from early developing retinas onward (22, 23). Loss-of-function studies have shown that Pou4f1 is mainly involved in RGC dendritic morphogenesis, although a modest reduction of RGC number (~30%) has also been observed in *Pou4f1^CKOAP/KO^* retina (24–28). Furthermore, Pou4f1 is sufficient to replace Pou4f2 in driving RGC developmental programs (29), and Pou4f1 was found to share synergistic functions with Pou4f2 in RGC development (27), suggesting that Pou4f1 can activate key regulatory genes for RGC differentiation and functions and part of these activities can be compensated by Pou4f2 in normal developmental program. Several recent studies have uncovered a number of Pou4f1 target genes in retina at P3, including transcription factors, transmembrane and intracellular structural molecules involved in RGC differentiation (26, 30, 31). Additionally, Pou4f1 was found to play a role in regulating the formation of contralateral RGCs by activating a subset of genes involved in axonal projection patterns (32). These data prompted us to investigate whether Pou4f1 plays a regulatory role on Tbr1-Jam2 expression and J-RGC formation (**Figure 1B**). In this report, we found that Pou4f1 is a direct upstream regulator for Tbr1 and Jam2 expression and J-RGC formation, establishing an epistatic relationship between Pou4f1, Tbr1, and Jam2 in the formation of J-RGCs.

## Materials and methods

### Animals

The generation and genotyping of *Six3-Cre, Tbr1^TauGFP-IRESCreERT2^ (Tbr1^tGFP^), Ai9, Jam2^CreER^, and Pou4f1^CKOAP^* mice were described previously (5, 9, 24, 33, 34). All mice were maintained on C57BL6/129 mixed backgrounds. Mouse lines of either sex at various ages were used. Pre-weaned animals were housed with their mother while weaned animals were housed in groups of no more than 5 in individually ventilated cages. All animal procedures followed the US Public Health Service Policy on Humane Care and Use of Laboratory Animals and were approved by the Animal Welfare Committee at The University of Texas Health Science Center at Houston (AWC-21-0102).

### Immunohistochemical analysis

Flat-mounted retinas were fixed with 4% paraformaldehyde (PFA; Electron Microscopy Sciences, Hatfield, PA), embedded in paraffin or OCT, and sectioned into 20 μm. Retinal sections or flat-mounted retinas were fixed with 4% PFA, then incubated with the primary antibodies for 3 days at 4°C. Primary antibodies used were chicken anti-GFP (1/1000 dilution, Cat #A10262, Thermo Fisher) and rabbit anti-Pou4f1 (1/500 dilution, Cat #ab245230, Abcam, Waltham, MA). Secondary antibodies conjugated with Alexa-488 and -555 (Life Technologies, Carlsbad, CA) were used in 1:800 dilution. DAPI (2.5 µg/ml, #D1306; Thermo Fisher Scientific, Waltham, MA) was used to stain nuclei. Images were captured using Zeiss LSM 780 or Zeiss LSM 800 confocal microscopes (Carl Zeiss, Thornwood, NY) and exported as TIFF files into Adobe Photoshop (Adobe Systems, San Jose, CA). Cell counting was conducted using the cell counter plugin of NIH ImageJ.

### Alkaline phosphatase staining

Alkaline phosphatase (AP) staining was conducted as previously described with minor modifications (24, 35). Whole eyeballs were fixed with 10% neutrally buffered formalin for 5 minutes. The retinas were removed and flat mounted on a piece of nitrocellulose membrane, post-fixed for 10 minutes at room temperature, washed twice in phosphate-buffered saline (PBS), and heated in PBS for 30 minutes in 65°C water bath to inactivate endogenous AP activity. AP staining was performed in AP staining solution (0.1 M Tris/pH 9.5, 0.1 M NaCl, 50 mM MgCl_2_, 0.34 g/ml p-nitroblue tetrazolium chloride, and 0.175 g/ml 5-bromo-4-chloro-3-indolyl phosphate) for 24-48 hours at room temperature with gentle shaking until dense color was developed in the dendrites, somas, and axons. After staining, retinas were washed 3 times for 5 minutes in PBS, post-fixed in PBS with 4% PFA briefly, dehydrated through an ethanol series, and then cleared with 2:1 benzyl benzoate/benzyl alcohol. Montages of the whole retina were acquired on a Zeiss Axio Imager 2 microscope equipped with a motorized xyz drive (Carl Zeiss, White Plains, NY).

### RNAscope *in situ* hybridization

ISH was performed using RNAscope technology with minor modifications (Advanced Cell Diagnostics, Minneapolis, MN) (36). Briefly, 9 µm paraffin or 10 μm cryo-sections mounted on Superfrost™ Plus glass slides were subjected to RNAscope 2.5 HD Detection Reagents-Brown kit (#322310) following manufacturer’s protocols. The procedure involved a 5-minute simmering in antigen retrieval reagents followed by RNAscope protease III for 30 minutes at 40°C. After washing twice in H_2_O, the sections were hybridized with RNAscope *in situ* probes for 2 hours at 40°C and processed according to the manufacturer’s protocols. According to ACD’s instructions, each mRNA molecule hybridized to a probe appears as a separate dot. The brown signal was examined and collected using an Olympus IX-70 inverted microscope. The probes used in this study was mouse Jam2-C1 (#467321).

### Terminal deoxynucleotidyl transferase dUTP Nick-End Labeling assay

An *in situ* cell death detection kit (Roche, Pleasanton, CA) was used for the TUNEL assay. DAPI (2.5 µg/ml) was used for nuclei staining.

### *In vivo* electroporation

Mice aged 2 to 3 months were anesthetized with a combination of ketamine and xylazine (94/5 mg/kg; IP). A small incision was created in the sclera with a 30-gauge needle. One µl of DNA solution (0.5-2 µg/µl) in 0.1x PBS containing 0.05% fast green was injected into vitreous using 34-gauge NanoFil^®^ system (World Precision Instruments, Sarasota, FL). After DNA injection, tweezer-w/horseshoe electrode (#CUY675P3, Bulldog Bio, Portsmouth, NH) was briefly soaked in PBS, then placed to hold the eyeball. Four 30 V square pulses (50 ms duration, 950 ms interval) were delivered *via* a square pulse electroporator NEPA21 (NEPAGENE, Chiba, Japan).

### CUT&Tag sequencing and data analysis

Four retinas isolated from wildtype mouse embryos at E16.5 were pooled, and then dissociated using papain dissociation system (#LK003150, Worthington Biochemical Corporation, Lakewood, NJ). The Cleavage Under Targets and Tagmentation (CUT&Tag) library was prepared using CUT&TAG-IT Assay Kit (#53160, Active Motif, Carlsbad, CA) following manufacturer protocol. Briefly, dissociated cells were washed with 1X wash buffer. Cells were then bound to Concanavalin A-coated magnetic beads. One μg of primary antibodies was applied to cell-bound beads and incubated overnight at 4°C. Primary antibodies used for the precipitation were: rabbit anti-Pou4f1 (#ab245230, Abcam, Waltham, MA), rabbit anti-Histone H3K9AC (#39017, Active Motif) and Rabbit IgG (#13-0042, EpiCypher, Durham, NC). Cell-bound beads were incubated with guinea pig anti-rabbit secondary antibody in Dig-Wash Buffer, subsequently with pA-Tn5 transposase in DIG-300 buffer at room temperature for 1 hour and then incubated in Tagmentation buffer at 37°C for 1.5 hours. Tagmented DNA fragments were extracted by incubating in PK buffer (16 mM EDTA, 0.1% SDS and 83.6 µg/ml Proteinase K) at 55°C for 1 hour, purified with the spin column, and amplified using indexed primers. The final libraries were submitted to Cancer Genomics Center at The University of Texas Health Science Center at Houston. The concentrations of the libraries were examined by Qubit 1xdsDNA HS Assay Kit (#Q33231, Thermo Fisher Scientific, Waltham, MA). The quality of the final libraries was examined using Agilent High Sensitivity DNA Kit (#5067-4626) by Agilent Bioanalyzer 2100 (Agilent Technologies, Santa Clara, CA), and the library concentrations were determined by qPCR using Collibri Library Quantification kit (#A38524500, Thermo Fisher Scientific). The libraries were pooled evenly and subjected to the paired-end 75-cycle sequencing on an Illumina NextSeq 550 System using High Output Kit v2.5 (#20024907, Illumina, San Diego, CA).

To analyze the CUT&Tag-seq data, sequence reads were trimmed free of adaptor sequences and masked for low-complexity or low-quality sequences, then mapped to the mouse mm10 reference genome using Bowtie2 (v2.4.5) software (37). Peak calling was performed by SEACR under relaxed mode (38). Two independent replicates using anti-Pou4f1 antibody and rabbit IgG, respectively, were analyzed using SEACR, and plotCorrelation (deepTools) was used to analyze the sample correlations *via* Pearson method (39). Data in **Figure 1** with the CUT&Tag-seq peaks that intersect with E16.5 scATAC-seq RGC-enriched peaks were conducted using bedtools (v2.30.0) (40). The raw datasets for each sample have been deposited in NCBI (Geo dataset: GSE221209). Enrichment analysis was conducted on Pou4f1-bound peaks using the enrichPathway and enrichKEGG tools, which are based on the REACTOME and KEGG databases, respectively (41, 42). Detailed information, including codes and vignettes, can be found in GitHub at https://github.com/YuLab-SMU/biomedical-knowledge-mining-book.

### Chromatin immunoprecipitation and quantitative PCR

ChIP assays were performed as previously described (43), with minimal modifications. Retinas were isolated from E16.5 wildtype embryos and were cross-linked with 1% formaldehyde for 10 minutes at RT, stopped by 0.125 M glycine and then homogenized in the cell lysis buffer (5 mM PIPES pH 8.0, 85 mM KCl, 0.5% NP40, and proteinase inhibitors). Nuclei were collected and resuspended in the nuclei lysis buffer (50 mM Tris-HCl pH 8.1, 10 mM EDTA, 1% SDS, and proteinase inhibitors). Chromatin was sheared by a Diagenode Bioruptor Plus Sonication system (Diagenode, Denville, NJ). Fragmented chromatin was precleared with 2.5 µg of normal rabbit IgG, then incubated overnight with 1 µg of rabbit anti-Pou4f1 antibody (#ab245230, Abcam) or normal rabbit IgG (#13-0042, EpiCypher). Antibody-bound chromatin complex was precipitated with salmon sperm DNA/Protein A agarose (EMD Millipore, Burlington, MA), then washed sequentially with RIPA (150 mM NaCl, 50 mM Tris-HCl pH 8.0, 0.1% SDS, 0.5% Deoxycholate, 1% NP40, and 1 mM EDTA), high-salt buffer (50 mM Tris-HCl pH 8.0, 500 mM NaCl, 0.1% SDS, 0.5% Deoxycholate, 1% NP40, and 1 mM EDTA), LiCl wash buffer (50 mM Tris-HCl pH 8.0, 1 mM EDTA, 250 mM LiCl, 1% NP40, and 0.5% Deoxycholate) and TE for 10 minutes each at 4°C. Cross-linking was reversed at 65°C overnight. DNA was extracted by phenol/chloroform, precipitated with ethanol, and dissolved in 30 µl of water. Three µl of DNA solution was used for one real-time, quantitative PCR (qPCR) reaction. To analyze specific Pou4f1-bound DNA, we performed qPCR using the CFX Connect Real-Time PCR Detection System with iTaq Universal SYBR Green SuperMix (#1725122, Bio-Rad, Hercules, CA). The qPCR primers are described below.

**Table:** 

Gene name	Primer sequence
Atoh7	Forward: 5- CCAACATCTGTCGCTCTGAA -3’ Reverse: 5’- AACACCACCACCCTGACTTC -3’
Pou4f2	Forward: 5’- TACAGGGTGAGCTGGGACTT -3’ Reverse: 5’- CAGCACATGCGCTCTGTATT -3’
Pou4f1	Forward: 5’- TCATGTAACACATTGCCCTGA -3’ Reverse: 5’- TTCCCACCTTAACCTTGCAC -3’
FoxP1	Forward: 5’- CTTTCGATTGCAGGGTAAGG -3’ Reverse: 5’- GACCCTGTGCTCAGTCCAGT -3’
Satb1	Forward: 5’- AAGGGGAGGAGGGAGAAACT -3’ Reverse: 5’- TCCGCAGCCTTCTGAGTTAT -3’
Satb2	Forward: 5’- TATTCCCACCAGCAGGACT -3’ Reverse: 5’- CATGGCCACTGAGAAGAACA -3’
Irx1	Forward: 5’- TCAGAACCTCAGGACGGAGA -3’ Reverse: 5’- TCATTCACACCGTTGCTGTT -3’
Cdh8	Forward: 5’- GCCAGCCTGATTTTCCATTA -3’ Reverse: 5’- GATGGCAGCTGTTAGCTTGG -3’
Sorcs3	Forward: 5’- TGGAGCAAAGCTTTTACATGG -3’ Reverse: 5’- TGTGGGTATTTCAGGTTTGCT -3’
Cartpt	Forward: 5’- TCAGGAAATCTCTGGCCATT -3’ Reverse: 5’- TGTGCCCTGTAGCCTTCTTT -3’

### Statistical analysis

All data are presented as mean ± standard deviation for each genotype. For all comparisons between ChIP-qPCR with anti-Pou4f1 antibody and normal IgG, a two-tailed, two-sample Student’s *t*-test in Excel (Microsoft, Redmond, WA) was used for measurements. Results were considered significant when P<0.05.

## Results

### CUT&Tag analysis uncovered Pou4f1-bound DNA elements in E16.5 retinas

To explore the potential role of Pou4f1 in RGC differentiation and subtype formation, we first performed a CUT&Tag sequencing analysis using a rabbit anti-Pou4f1 antibody (**Supplementary Figures 1A–C**) and mouse embryonic 16.5 (E16.5) retinal cells to generate barcoded PCR libraries that are enriched for Pou4f1-mediated binding (64). In parallel, rabbit IgG was used as a negative control for peak calling analysis, and rabbit anti-H3K9AC antibody was used to mark active enhancers and promoters. The libraries were subsequently sequenced to obtain pair-ended (PE75) sequencing data for downstream bioinformatics analysis (detailed described in the Material and Methods). From a set of replicate experiments (**Figure 1A**), we found the two datasets are highly correlated on their mapped read counts across the genome (Pearson correlation coefficient = 0.8, **Supplementary Figure 1D**), indicating that the data are reproducible. Using SEACR peak calling method, which was designed for calling peak from sparse background (38), we identified 8,032 Pou4f1-enriched regions/peaks (**Supplementary Table 1**). These peaks are distributed mainly within and flanking the gene bodies, including promoters, exons, and introns, and to a lesser extent in the intergenic regions (upper panel, **Figure 1B**).

The high peak number was unlikely caused by experimental variation because the datasets from replicate experiments were highly correlated. The top enriched DNA-binding motif identified by HOMER was consistent with known Pou4f1-binding sites, although the motif was only identified in 9.8% of all targets examined (lower panel in **Figure 1B**). These data suggest that Pou4f1 is likely involved in a wide array of cellular processes during RGC differentiation, and Pou4f1 may bind to many of these elements indirectly through physically interacting with other factors. Consistent with this notion, when this long list of 8,032 binding sites was intersected with an RGC-specific open chromatin dataset identified by a multiomic snRNA-seq coupled with snATAC-seq on E16.5 retinas (Kiyama and Mao, unpublished results), we found that the number of Pou4f1-bound open chromatin regions was reduced to 2604 (**Supplementary Table 2**, hereinafter labeled as “Pou4f1-BOC” standing for Pou4f1-bound open chromatin), and HOMER motif analysis on this shorter list identified Pit1/Oct1-binding motif as the highest matched motif distributed in 47.8% of these targets (**Figure 1C**, upper panel). Because the predicted Pit1-binding site and Pou4f1 binding site are highly similar (**Supplementary Figure 1E**), confirming these are bona fide Pou4f1-bound elements in E16.5 RGCs.

Next, we used *ChiPseeker* R package to map and annotate Pou4f1-BOC associated genes, and then used the *enrichPathway* and *enrichKEGG* functions of *clusterProfiler* R package to identify biological processes and functional categories of genes with Pou4f1-BOC sites (41, 44). The enrichment pathway analysis of genes with Pou4f1-BOC retrieved biological processes involved in neuronal system, synapse transmission, and axonal guidance (**Figure 1D**). By KEGG analysis, Pou4f1-BOC were found near genes involved in several signaling pathways, axonal guidance, and synapses (**Figure 1E**). Together, these analyses exposed the previously unknown, complex functions of Pou4f1 in RGC differentiation and function.

### Pou4f1 occupies DNA elements in close proximity to genes critical for RGC development

**Figure 2:**
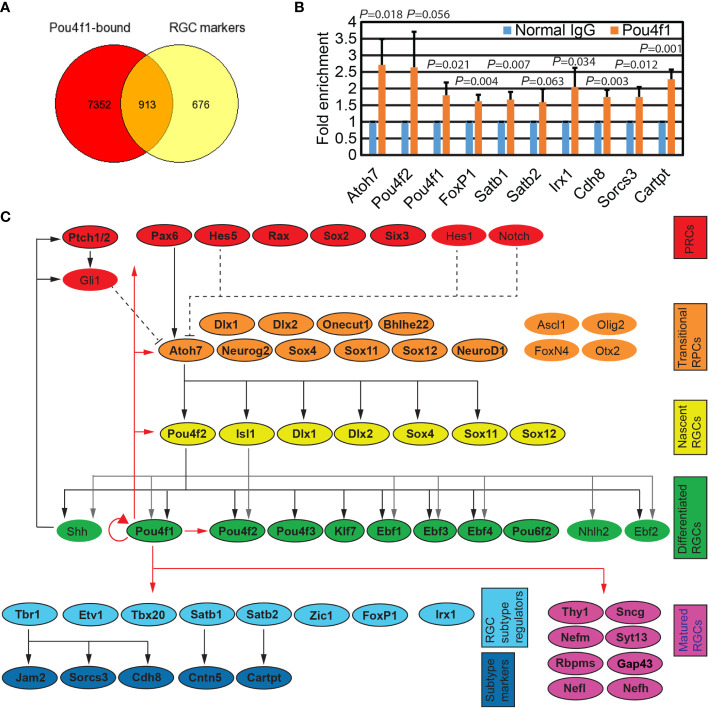
Pou4f1 binds to key regulators for RGC development. **(A)** Venn diagram depicting the overlap of genes associated with Pou4f1-bound elements displayed in **Figure 1B** and RGC-enriched genes from our E16.5 scRNA-seq dataset. **(B)** Quantitative ChIP-PCR validation of a selective subset of Pou4f1-bound peaks. **(C)** Diagram illustrating the known genetic regulatory network (GRN) in RGC development. Genes are categorized according to their involvement in development from retinal progenitor cells (RPCs) to matured RGCs. Genes harboring Pou4f1-enriched peaks near their gene bodies are highlighted in colored boxes and indicated with bold letter. The known GRN hierarchic edges are indicated with black or gray arrow and black dotted lines, and the novel GRN hierarchic edges are indicated with red lines.

We next compared the genes containing Pou4f1-bound sites with genes that are enriched in RGC clusters from single cell RNA-seq data (**Supplementary Table 3**, and unpublished snRNA-seq data from Kiyama and Mao). As expected, we found more than half of the genes enriched in RGC clusters harbored Pou4f1 peaks in or near their gene bodies (**Figure 2A**). Interestingly, we also found many Pou4f1 peaks located in genes enriched in non-RGC cells, including naïve retinal progenitor cell (nRPC) and transitional RPCs (**Supplementary Figure 2**). To quantitatively validate Pou4f1 CUT&Tag dataset, we performed ChIP-qPCR analysis on a small, selective subset of the Pou4f1-targeted elements that contain predicted Pou4f1-binding motif, and found that ChIP-qPCR analysis were consistent with the CUT&Tag dataset (**Figure 2B**). To better visualize some of the key target genes in retinas and, hence, the inferred functions of Pou4f1 through these targets, a simplified gene regulatory network (GRN) for RGC development was created, according to the relevant literature (5, 7, 8, 17, 19, 20, 22–24, 45–47), and a number of Pou4f1-bound genes encoded for transcription factors and well-known RGC differentiation markers were color-coded and mapped to GRN at different hierarchical levels according to their roles in development (**Figure 2C** and **Supplementary Figure 2**) (4, 48–51). This simplified Pou4f1-interacted GRN revealed many previously defined functions of Pou4f1, such as a well-established auto-regulatory function of Pou4f1 (52), and its direct involvement in regulating RGC marker expression and subtype formation. It also uncovered a possible feedback regulatory loop through which Pou4f1 controls the upstream regulators, such *as Atoh7, Pou4f2, and Isl1*, which require further investigation. In the next sections, we focused on elucidating Pou4f1’s function on RGC subtype formation.

### Pou4f1 is required for the expression of Tbr1-Jam2 and the differentiation of J-RGCs

**Figure 3:**
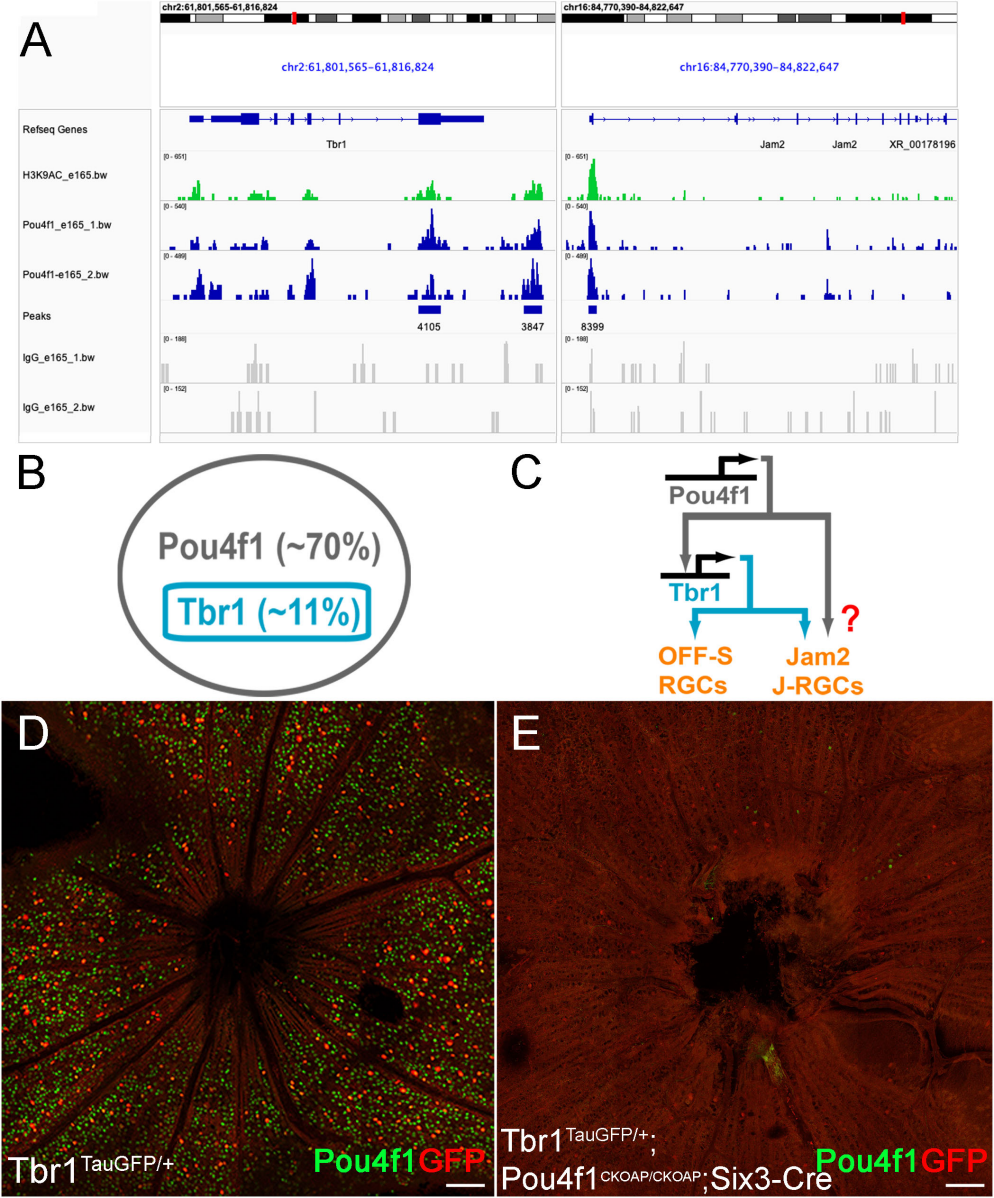
Pou4f1 is essential for *Tbr1* expression. **(A)** Pou4f1-enriched CUT&Tag peaks found within and near *Tbr1 and Jam2* genomic loci. **(B)** Schematic illustration showing the relationship of Tbr1+ RGCs and Pou4f1+ RGCs. **(C)** Transcriptional cascade of Pou4f1, Tbr1 and Jam2 hypothesized by Pou4f1 CUT&Tag sequencing analysis. **(D, E)** Flat-mounted immunostaining using anti-GFP (red) and Pou4f1 (green) antibodies on *Tbr1^tGFP/+^* **(D)** and *Tbr1^tGFP/+^; Pou4f1^CKOAP/CKOAP^; Six3-Cre* **(E)** retinas. Scale bar: 100 μm.

Among the 8,032 enriched regions, we first focused on the *Tbr1-Jam2* regulatory pathway. We found that Pou4f1-bound regions are enriched in both Tbr1 and Jam2 loci (**Figure 3A**). In *Tbr1* locus, we detected two Pou4f1 peaks, including one in exon 6 (chr2: 61811552-61812426) and one in a region slightly downstream to the 3’ UTR (chr2: 61815651-61816336) (**Figure 3A**). Interestingly, both regions encompassed several DNase hypersensitive sites across ENCODE samples, which were validated by high H3K4me3, H3K27ac and/or CTCF ChIP-seq signals and, hence, designated as candidate *Cis*-Regulatory Elements (cCREs; ENCODE Accession: EM10E0697073 to EM100697075 and EM10E0697083–EM10E0697085 respectively) (53), suggesting these regions may serve as enhancers for *Tbr1* transcription activation. In *Jam2* locus, we detected one Pou4f1-bound peak flanking the promoter and first exon (chr16: 84774016-84775100) (**Figure 3A**). Similarly, three cCREs (EM10E0627570 to EM10E0627572) are found encompassed in this region, implicating that this region is critical for *Jam2* expression. Coincidently, a Tbr1 ChIP-seq experiment on P2 mouse cortical neurons has also identified a Tbr1-bound element in this region (chr16:84774081- 84774541) (54). In addition, Pou4f1 peak was also found in Tbr1 downstream gene *Sorcs3* locus (promoter and exon 1: chr19:48205984-48206850), partially overlapped with a Tbr1-bound element found in P2 cortical neurons (chr19:48,205,029-48,206,269) (**Supplementary Figure 2**) (54). Because Tbr1 is exclusively expressed in Pou4f1-expressing RGCs and is essential for the formation and dendritic morphogenesis of J-RGCs (**Figure 3B**) (5, 21), and *Sorcs3* has been shown to be an effector gene involved in dendritic morphogenesis for J-RGCs (21), raising a possibility that Pou4f1 is an upstream regulator for *Tbr1-Jam2* expression and Tbr1-expressing J-RGCs (**Figure 3C**).

To explore whether Pou4f1 plays a role in regulating *Tbr1* and *Jam2* expression, we bred *Six3-Cre* (an embryonic retina-specific Cre line) with *Pou4f1^CKOAP/CKOAP^* to delete *Pou4f1* in early embryonic retinas. We first generated P7 *Six3- Cre : Tbr1^TauGFP/+^:Pou4f1^CKOAP/CKOAP^* animals and their *Tbr1^TauGFP/+^* littermates as positive controls, and conducted immunofluorescent (IF) staining for Pou4f1 and GFP. We found that the number of GFP-expressing Tbr1-expressing RGCs was significantly down- regulated in *Six3-Cre : Tbr1^TauGFP/+^:Pou4f1^CKOAP/CKOAP^* retinas compared to *Tbr1^TauGFP/+^* control retinas (459 in control *vs.* 58 in mutant; compared **Figures 3D, E**), suggesting that *Pou4f1* is essential for the formation of a large fraction of Tbr1^+^ RGCs. In contrast to this finding, a previous study has shown a 45% down-regulation of *Tbr1* expression levels in P3 *Pou4f1*-deleted cells (**Supplementary Figure 3A**). This discrepancy prompted us to investigate the presence of Tbr1-expressing RGCs in *Pou4f1*-deleted retinas compared to *wildtype* retinas. We conducted immunofluorescence (IF) staining for Tbr1 expression in P7 *Pou4f1^CKOAP/+^* and *Six3-Cre : Pou4f1^CKOAP/CKOAP^* retinas and observed a decrease in the number of Tbr1-expressing RGCs in *Six3-Cre : Pou4f1^CKOAP/CKOAP^* retinas, although the difference was not as significant as that revealed by Tbr1-driven GFP expression (418 in control *vs.* 207 in mutant; see **Supplementary Figures 3B, C**). These findings suggest that Pou4f1 is likely involved in the formation of Tbr1-expressing RGCs as well as in regulating Tbr1 expression levels.

**Figure 4:**
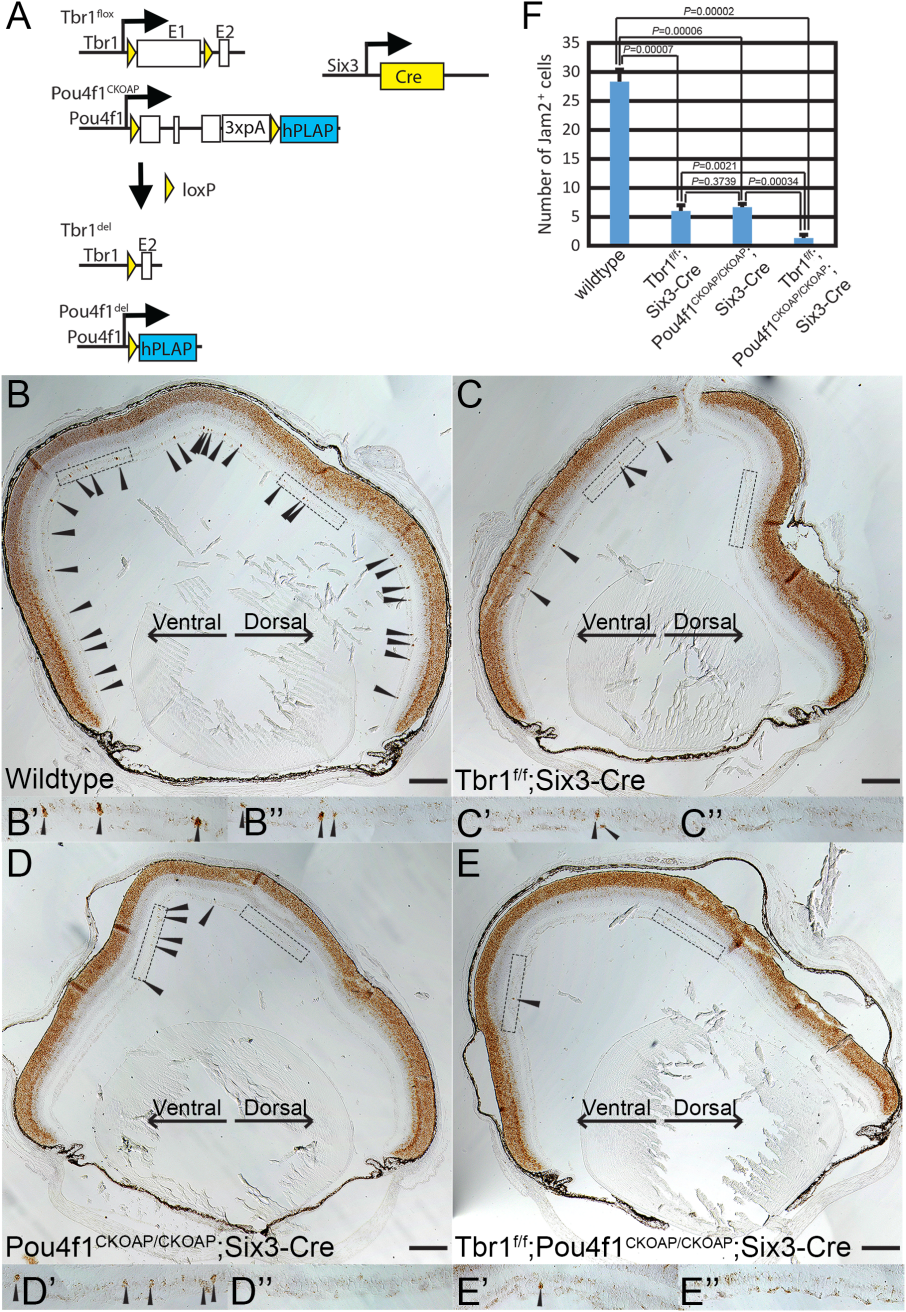
Pou4f1 is essential for *Jam2* expression. **(A)** Schematic illustration showing Tbr1-flox, Pou4f1-CKOAP and Tbr1-Pou4f1-double CKO by Six3-Cre. **(B-E)** *In situ* hybridization (ISH) of *Jam2 on* P7 wildtype **(B)**, *Tbr1^f/f^; Six3-Cre* **(C)**, *Pou4f1^CKOAP/CKOAP^*; *Six3-Cre* **(D)** and *Tbr1^f/f^; Pou4f1^CKOAP/CKOAP^; Six3-Cre* **(E)** retinal sections. (B’-E’) ISH images of the ventral part of the retinas. (B’’-E’’) ISH images of the dorsal part of retinas. **(F)** Quantification and statistical analysis of ISH data in panels **(B-E)**. Scale bar: 200 μm.

Because Tbr1 is essential for *Jam2* expression and the formation of Jam2-expressing J-RGCs, we further bred *Six3-Cre : Tbr1^f/+^:Pou4f1^CKOAP/+^* with *Tbr1^f/f^:Pou4f1^CKOAP/CKOAP^* to generate *Tbr1^f/+^:Pou4f1^CKOAP/+^* (WT control), *Six3-Cre : Tbr1^f/f^*, *Six3-Cre : Pou4f1^CKOAP/CKOAP^* and *Six3-Cre : Tbr1^f/f^:Pou4f1^CKOAP/CKOAP^* animals (**Figure 4A**), and then conducted RNAscope *in situ* hybridization (ISH) for *Jam2* expression. Consistently, we found that *Jam2* expression is down-regulated to basal levels in both *Six3-Cre : Tbr1^f/f^* and *Six3-Cre : Pou4f1^CKOAP/CKOAP^* retinal sections compared to the control (WT: 28.33 ± 2.08, *Six3-Cre:Tbr1^f/f^*: 6.00 ± 1.00, *Six3-Cre:Pou4f1^CKOAP/CKOAP^*: 6.66 ± 0.57, *Six3-Cre:Tbr1^f/f^:Pou4f1^CKOAP/CKOAP^*:1.33 ± 0.57; **Figures 4B–F**). Furthermore, in *Six3-Cre : Tbr1^f/f^:Pou4f1^CKOAP/CKOAP^* double mutant retinas, *Jam2* expression seemed to be slightly reduced in comparison to *Tbr1-* or *Pou4f1*-single mutant, although the reduction is relatively modest (**Figures 4B–F**).

**Figure 5:**
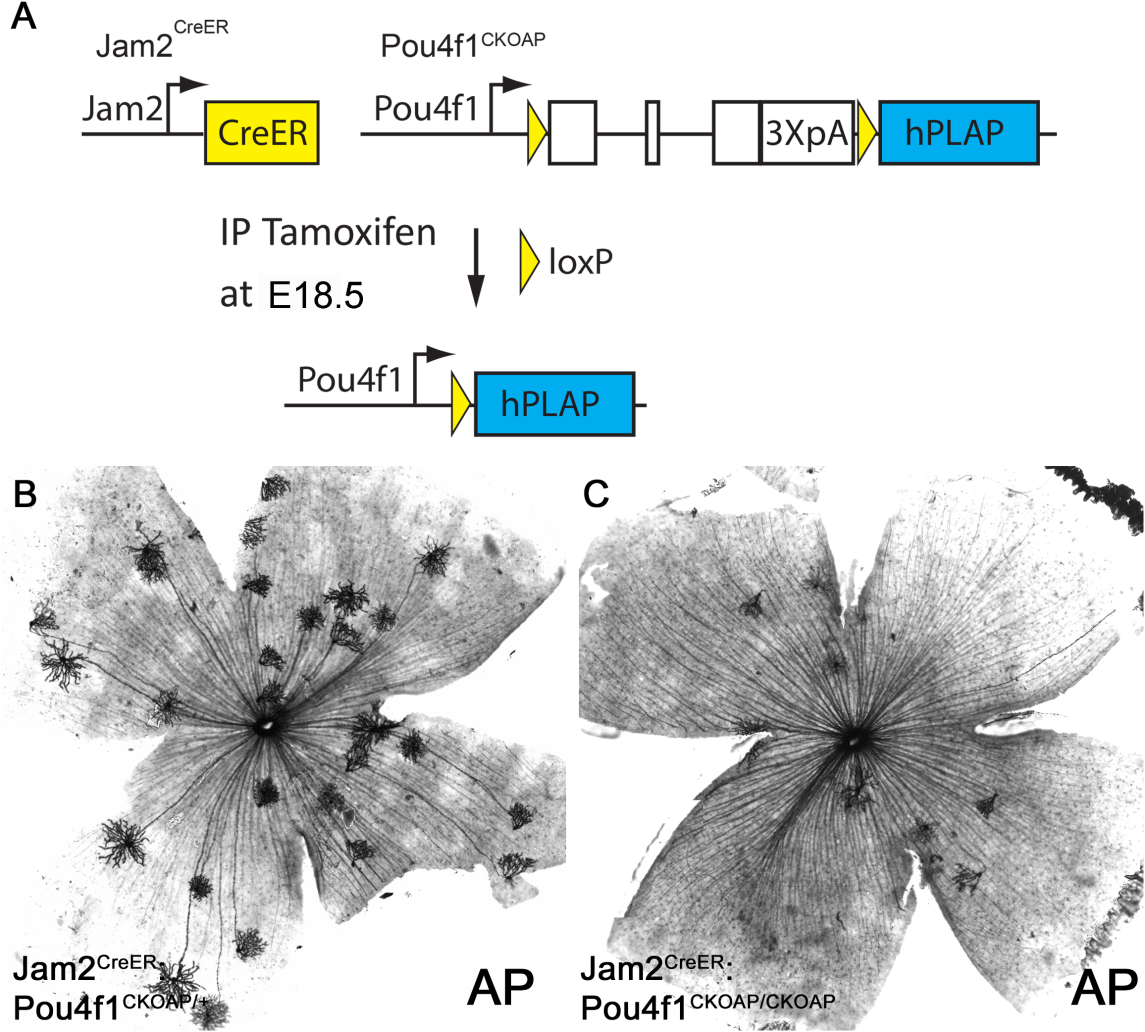
Pou4f1 is essential for J-RGC formation. **(A)** Schematic illustration of Pou4f1 knockout by *Jam2^CreER^*. **(B, C)** AP staining on *Jam2^CreER^; Pou4f1^CKOAP/+^* **(B)** and *Jam2^CreER^; Pou4f1^CKOAP/CKOAP^* **(C)** flat-mounted retinas.

The down-regulation of *Tbr1* and *Jam2* expression in *Pou4f1*-mutant retinas suggested that *Pou4f1* is required for the expression of *Tbr1* and *Jam2* and the formation of J-RGCs. To directly test this idea, we used genetic sparse labeling. We bred *Jam2^CreER ^: Pou4f1^CKOAP/+^* mice with *Pou4f1^CKOAP/CKOAP^* mice and induced Cre activity at embryonic day 18.5 (E18.5) by intraperitoneal injection of tamoxifen (**Figure 5A**). We isolated retinas from P30 *Jam2^CreER ^: Pou4f1^CKOAP/+^* and *Jam2^CreER ^: Pou4f1^CKOAP/CKOAP^* littermates for alkaline phosphatase (AP) staining. Consistent with the IF and ISH data, we found a significant reduction in the number of J-RGCs in Pou4f1-mutant retinas compared to controls (control: 18.75 ± 6.4, mutant: 8.00 ± 2.16, P = 0.04; **Figures 5B, C**). Furthermore, to determine whether Pou4f1 deletion leads to cell death in embryonic retinas, we performed a TUNEL assay on E18.5 wildtype and *Pou4f1^del/del^* retinal sections and observed TUNEL signal dispersed in the ganglion cell layer (GCL) throughout the peripheral and central retina (**Supplementary Figures 4A, B**). A 1.54-fold increase in TUNEL+ cells was detected in the GCL of *Pou4f1^del/del^* retina (28.17 ± 9.37; see **Supplementary Figures 4B**, **6D**) compared to wildtype (18.33 ± 7.97; see **Supplementary Figures 4A, C**). Taken together, these data substantiate our hypothesis that the *Pou4f1-Tbr1-Jam2* genetic hierarchy is the primary pathway for J-RGC subtype development.

### Pou4f1-bound elements possess subtype RGC-specific enhancer activity

**Figure 6:**
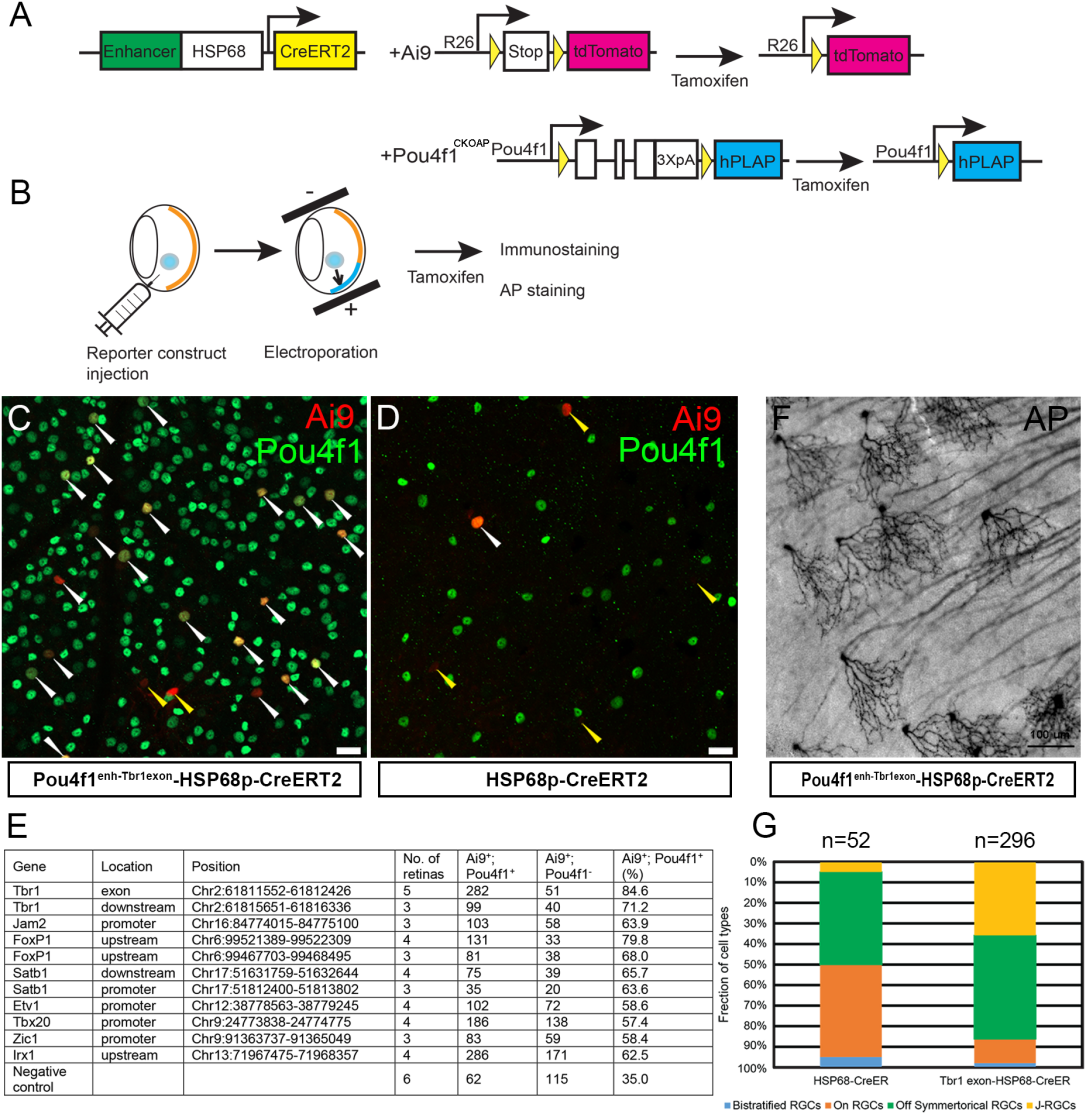
Pou4f1-bound elements as functional enhancers in RGC subtypes. **(A)** Schematic illustration showing the reporter system driven by Pou4f1-mediating enhancer. **(B)** Experimental strategy of enhancer assay. **(C, D)** Ai9 reporter expression driven by Pou4f1^Enh-Tbr1exon6^HSP68p-CreERT2 reporter **(C)** or negative control construct (HSP68p-CreERT2). **(D)** Ai9 and Pou4f1 co-expressing RGCs were indicated with white arrowheads; Ai9^+^ cells without Pou4f1 expression were indicated with yellow arrowheads. **(E)** Summary of enhancers assay. **(F)** Representative AP-stained image with Pou4f1^Enh-Tbr1exon6^HSP68p-CreERT2 reporter construct. **(G)** The fraction of cell types driven by Pou4f1^Enh-Tbr1exon^HSP68p-CreERT2 reporter and the negative control construct (HSP68p-CreERT2). Scale bars: 20 µm in **(C, D)** and 100 µm in **(F)**.

Differential gene expression is preceded and marked by the interaction between key TFs and enhancer elements to safeguard precise spatiotemporal expression patterns and quantitative dynamics of target genes. The binding of Pou4f1 to *Tbr1* and *Jam2* loci at E16.5 preceded the onset of *Tbr1* and *Jam2* expression in RGCs yet Pou4f1 is required for *Tbr1* and *Jam2* expression at postnatal stages suggested that Pou4f1-bound elements identified in E16.5 developing RGCs may serve as subtype-specific enhancer elements in mature RGCs. To test this idea, we selected a subset of Pou4f1-bound elements near genes with known functions in RGC subtype formation and cloned these fragments upstream to a HSP68-basal promoter fused to CreERT2-pA reporter construct (**Figure 6A**). A reporter construct without any Pou4f1-bound element was used as a negative control. We injected these constructs into adult Ai9 mouse retinas followed by a mild electroporation procedure and tamoxifen induction, and then isolated retinas 7 days later for IF staining for Pou4f1 and Ai9 expression analysis (**Figure 6B**).

In the retinas electroporated with Pou4f1^enh-Tbr1exon^-HSP68p-CreERT2 construct, 84.6% of the Ai9+ cells were Pou4f1+ RGCs (**Figures 6C, E** and **Supplementary Figure 5A**); whereas in retinas electroporated with control HSP68p-CreERT2 construct, only 35% of the Ai9+ cells were Pou4f1+ RGCs (**Figures 6D, E**). These data suggested that the 875 bp Pou4f1-bound element in the sixth exon of Tbr1 possessed high levels of enhancer activity directing reporter gene expression in Pou4f1-expressing RGCs. Similarly, a Pou4f1-bound element in the upstream region of Foxp1 also possessed high levels of enhancer activity in Pou4f1+ RGCs (**Figure 6E** and **Supplementary Figure 5B**). In addition, other elements were capable of directing expression of Ai9 reporter gene in Pou4f1+ RGCs, although the levels of correlation were not as high as the enhancer elements in Tbr1 exon 6 and Foxp1 upstream region (**Figure 6E** and **Supplementary Figures 5C–F**).

The Pou4f1-bound element in exon 6 of Tbr1 is located in a protein encoding region, which is conserved among species. It has been found to be part of a super-enhancer in mouse cortex (55). The highly correlated expression of Ai9+ signal and Pou4f1+ RGCs in Pou4f1^enh-Tbr1exon^-HSP68p-CreERT2 construct prompted us to test whether this element can function as subtype-specific enhancer. To test this, we injected Pou4f1^enh-Tbr1exon^-HSP68p-CreERT2 plasmid into adult *Pou4f1^CKOAP/+^* retinas followed by electroporation and tamoxifen induction, and isolated retinas 7 days later for AP staining analysis (**Figures 6A, B**). In the retinas electroporated with Pou4f1^enh-Tbr1exon^-HSP68p-CreERT2 construct, approximately 36% of the AP+ RGCs appeared as J-RGCs (**Figures 6F, G**), whereas in retinas electroporated with negative control construct, less than 4% of the AP+ RGCs were J-RGCs (**Figure 6G**). These data indicated that this 875 bp element was capable of conferring high-level enhancer activity, not just in Pou4f1-expressing RGCs but also, preferentially, in J-RGCs.

## Discussion

Pou4f1 expression in differentiated RGCs was identified nearly three decades ago. However, its functions in regulating RGC development were only partially revealed. In E16.5 mouse retinas, the number of Pou4f1-expressing RGCs accounts for approximately 5% of all retinal neurons (**Supplementary Figure 1C**, and Kiyama and Mao unpublished data), hindering the effort to uncover Pou4f1’s genome occupancy by conventional ChIP-seq analysis, which requires millions of cells as starting materials. With the advance of the CUT&Tag sequencing technique, we can bypass the need of large number of cells and have identified Pou4f1’s *in vivo* binding sites. By mapping to RGC-specific open chromatin, we can distinguish Pou4f1-bound elements in open and close chromatin, respectively, critical in understanding how Pou4f1 binds and acts on its various targets along development.

### Extensive Pou4f1-to-chromatin interaction revealed by CUT&Tag

The extensive long list of 8,032 Pou4f1-bound elements identified in this study is less likely due to experimental artifact because: 1) the dataset was obtained from two highly correlated replicate experiments and Homer analysis revealed that the most enriched binding motif in our list matched well to Pou4f1 binding motif; and 2) the CUT&Tag procedure contains several washing steps using high salt concentration (150-300 mM NaCl), which is not favorable for weaker protein-DNA interaction. Additionally, we have uncovered many previously known Pou4f1 target genes and Pou4f1-bound elements.

On the list of Pou4f1-BOC, where the 8,032 Pou4f1-bound sites were intersected with RGC-specific open chromatin regions, we found that 47.18% of the Pou4f1-BOC elements contained predicted Pou-TF binding motif (**Figure 1C**). In contrast, within the other Pou4f1-bound elements mapped to the close chromatin regions in RGCs, only 3% contained predicted Pou-TF binding motif. This contrast suggested that Pou4f1 likely binds to its DNA targets through direct Pou4f1-to-DNA interaction when the targets are in an “open” chromatin state, and its binding to “close” chromatin structure is most likely through indirect interaction with other TFs and/or epigenetic factors. The factors involved in such indirect interaction are yet to be identified.

Another interesting feature revealed by Homer analysis on Pou4f1-BOC is that the predicted Isl1 binding motif was revealed as the second-most abundant motif (15.90%) (**Figure 1C**). It has been shown that Pou4f2 and Isl1 physically interact, forming a complex to exert its transcription activity in mouse RGCs, and the cognate Pou4-like factors and Isl1-like TFs genetically interact with each other in regulating touch neuron development in *C. elegans* (19, 56–58). Our data implicated that Isl1 may also interact with Pou4f1 in a similar manner to convey transcription activity on some of the RGC genes, although this notion remains to be determined.

### Multiple roles of Pou4f1 in RGC transcription networks

Through genetic loss-of- and gain-of-function studies, the “Atoh7→Pou4f2/Isl1→Pou4f/other TFs” transcriptional cascade is well established as the main pathway for RGC development (16, 17, 19, 45, 56). Atoh7 operates in post-mitotic RPCs to provide competency state favored RGC production. Pou4f2 and Isl1 are immediate downstream regulators of Atoh7 working together in early RGCs, and Pou4f1 and other TFs function downstream of Pou4f2/Isl1 in differentiated RGCs to maintain RGC functional specificity, survival, and subtype identity. Our CUT&Tag data suggested Pou4f1 may have more complex functions in this simplified lineal cascade.

First, we found that Pou4f1 binds to many genes encoding upstream regulator in RGC transcriptional network, such as Pax6, Rax, Atoh7, Pou4f2, and Isl1 (**Figure 2C**). In many developmental systems, feedback loop is a common mechanism to control the numbers of cells produced through development into a mature tissue/organ composed of properly balanced cell types, and TFs are the intrinsic elements in the cells to carry out this task. Our finding of Pou4f1-bound elements in many upstream regulators, which are activated in RPCs but not expressed in RGCs, suggests a possible role of Pou4f1 in silencing some of these genes in RGCs to prevent their aberrant expression in the wrong cells, which may lead to unwanted effects. Consistent with this notion, Pou4f1 has been shown to bind to and repress the expression of Neurod1 and Neurod4 in embryonic trigeminal ganglia (52). Conversely, a single transcription factor is unlikely solely responsible for negative feedback regulation. For instance, removing Pou4f2 and Isl1 do not cause a dramatic difference in chromatin status in RGCs (47, 59). The functional significance of Pou4f1 binding to these upstream genes remains to be elucidated.

Second, we found extensive Pou4f1 occupancy on its own locus, suggesting an auto-regulatory loop by Pou4f1. Transcriptional autoregulation is a common mechanism to stabilize the production of the transcription factor in a steady state. It is not surprising that Pou4f1 regulates its own expression through development into mature RGCs because Pou4f1 is turned on early in differentiated RGCs and stays activated in approximately 70% of all mature RGCs. Pou4f1 has been shown to auto-regulate its own expression in several sensory systems (52, 60). Our finding in E16.5 retinas resonates well with these studies.

We have also identified Pou4f1-bound sites on many RGC-enriched genes (**Supplementary Table 3**). For example, Sncg, Syt13, Gap43, Rbpms, and many others, are bound by Pou4f1 (**Supplementary Figure 6**). In many of these RGC-enriched genes, Pou4f1-bound elements are located within the open chromatin, which are also marked with H3K9AC binding, suggesting that Pou4f1 functions as an activator in maintaining the expression of these genes in differentiated RGCs. Consistent with our finding, a recent study identified a Pou4f1 binding site (5’-ATCAATATTTCATCT-3’) in the promoter of Sncg, which is capable of conveying Pou4f1-dependent enhancer activity in HEK293 cells (32) and, not surprisingly, this element is located in our Pou4f1-bound element (Chr14:34374429-34375186) (**Supplementary Table 1**). Furthermore, deleting Pou4f1 in RGCs leads to profound defect in the morphologies and numeric number of RGCs (28) and down-regulation of Sncg (Takae and Mao, manuscript in preparation). In addition, several well-studied, Pou4f1-dependent RGC-enriched genes, including Rbfox1, Eml1, Hpca, Mapk10, Snap91, Tusc5, Elfn1, Grm4, Pnkd, Rims1, Nptx1, Nptx2, Sez6l2, Cdh4, and Tmem25, identified in post-natal day 3 retinas (31), are found to be direct targets of Pou4f1 in this study (**Supplementary Figure 6**). Most of the Pou4f1-bound sites in these loci are localized in the open chromatin structure (**Supplementary Table 3**), suggesting Pou4f1 has already functioned on trans-activating these genes as early as E16.5.

### Pou4f1 involvement in RGC subtype formation

An intriguing finding in this study is the discovery of Pou4f1-bound elements within or in close proximity to several genes involved in RGC subtype development, including Tbr1, Jam2, Sorcs3, Foxp1, Satb1, Satb2, Irx1, Tbx20, and Zic1. Among these, we found that *Tbr1 and Jam2* expression are significantly down-regulated in Pou4f1-mutant retinas, placing them as direct down-stream targets of Pou4f1 during RGC development. We also showed that Pou4f1-bound region in exon 6 of Tbr1 is capable of conferring high levels of enhancer activity in J-RGCs. Together, our data established a Pou4f1-Tbr1 transcriptional cascade important for the development of J-RGCs.

In an attempt to understand ipRGC development, we have identified Irx1 and Tbx20 as downstream effector genes of Tbr2 for the development of a subset of ipRGC subtypes. It is known that Pou4f1 is not expressed in ipRGCs (5, 7), and Irx1 expression has been shown to be up-regulated in Pou4f1-mutant retinas (61), suggesting that Pou4f1 may play a role in suppressing Irx1 expression level in fate-undetermined RGC precursors, a plausible mechanism used to ensure subtype segregation and divergence during RGC development (62).

## Summary

The invention of novel genomic techniques has greatly advanced our understanding of how TFs and epigenetic factors function *in vivo* (63–65). We applied CUT&Tag- sequencing to identify genome occupancies of Pou4f1 in developing mouse retinas. A similar recent study has also identified genome occupancies for several key TFs, including Atoh7, Isl1, and Pou4f2, in RGC development (47, 59). The cross comparison between these target elements will further assist our understanding of how combinatorial TFs function during RGC differentiation. Moreover, the incorporation of these novel techniques into droplet-based, single-cell platforms to profile chromatin landscapes or TF occupancies in single cells will, inevitably, revolutionize how we view the interplay of different transcription factors and networks in time and space during development (66, 67).

## Data availability statement

The datasets presented in this study have been deposited in the NCBI Geo datasets under the accession number GSE221209.

## Ethics statement

The animal study was reviewed and approved by the Animal Welfare Committee at The University of Texas Health Science Center at Houston (AWC-21-0102).

## Author contributions

CAM, TCB, TK, and HYA designed the experiments. CAM, TK, HYA, and TCB executed the experiments. CAM wrote the paper. All authors contributed to the article and approved the submitted version.
