# Supplementary figures and images for: Pou4f1-Tbr1 transcriptional cascade controls the formation of Jam2-expressing retinal ganglion cells
Source: Front Ophthalmol (Lausanne). 2023 May 18;3:1175568. doi: 10.3389/fopht.2023.1175568 (PMC10926710; doi:10.3389/fopht.2023.1175568)

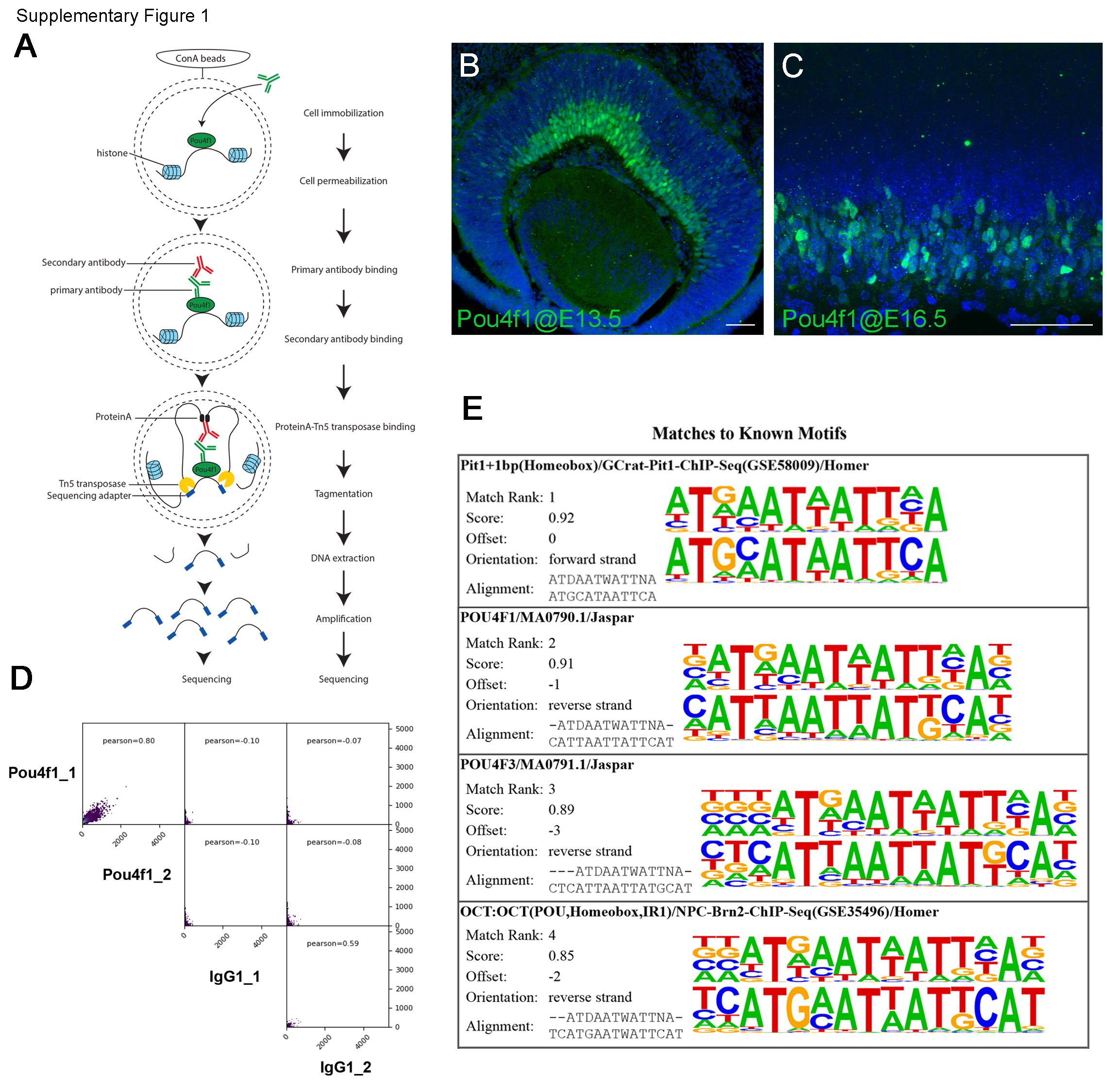

Supplement: Supplementary file 1 [file Image_1.tiff]

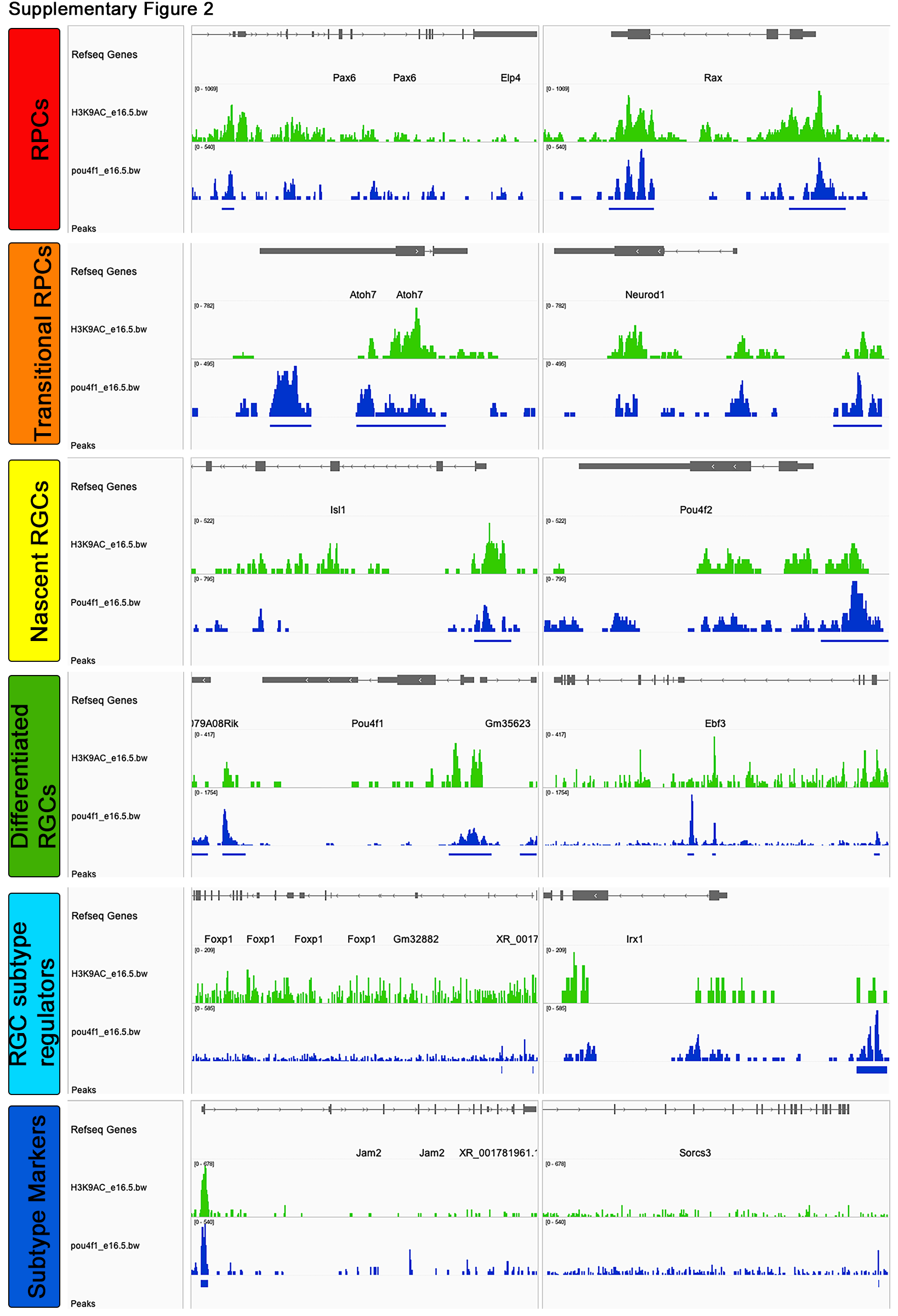

Supplement: Supplementary file 2 [file Image_2.tif]

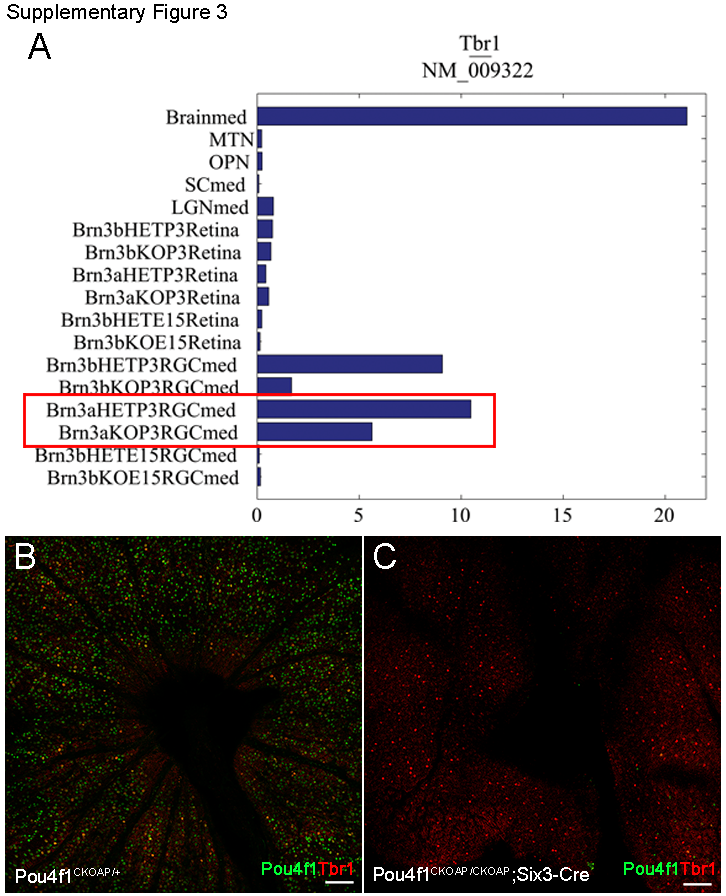

Supplement: Supplementary file 3 [file Image_3.tif]

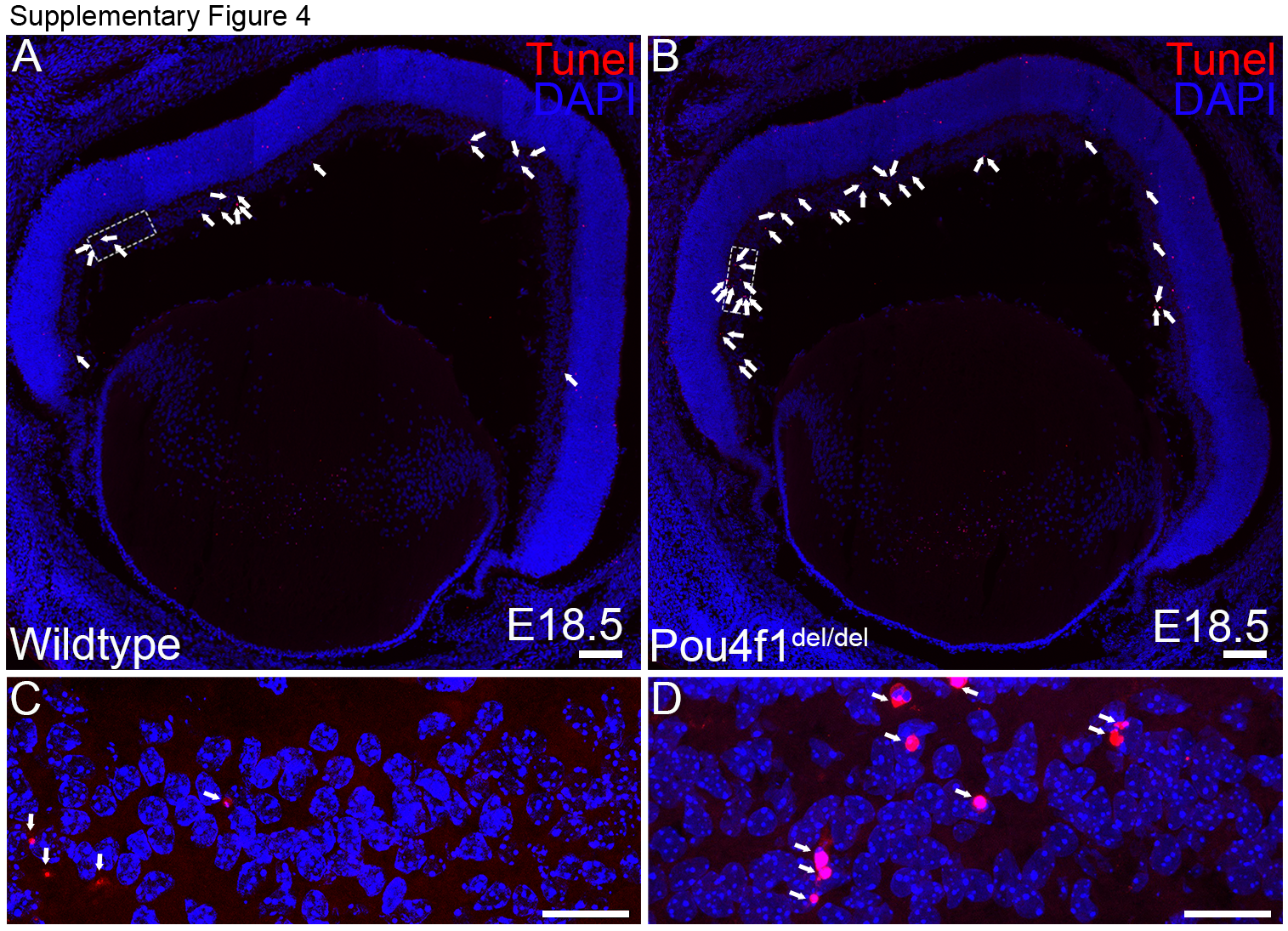

Supplement: Supplementary file 4 [file Image_4.tif]

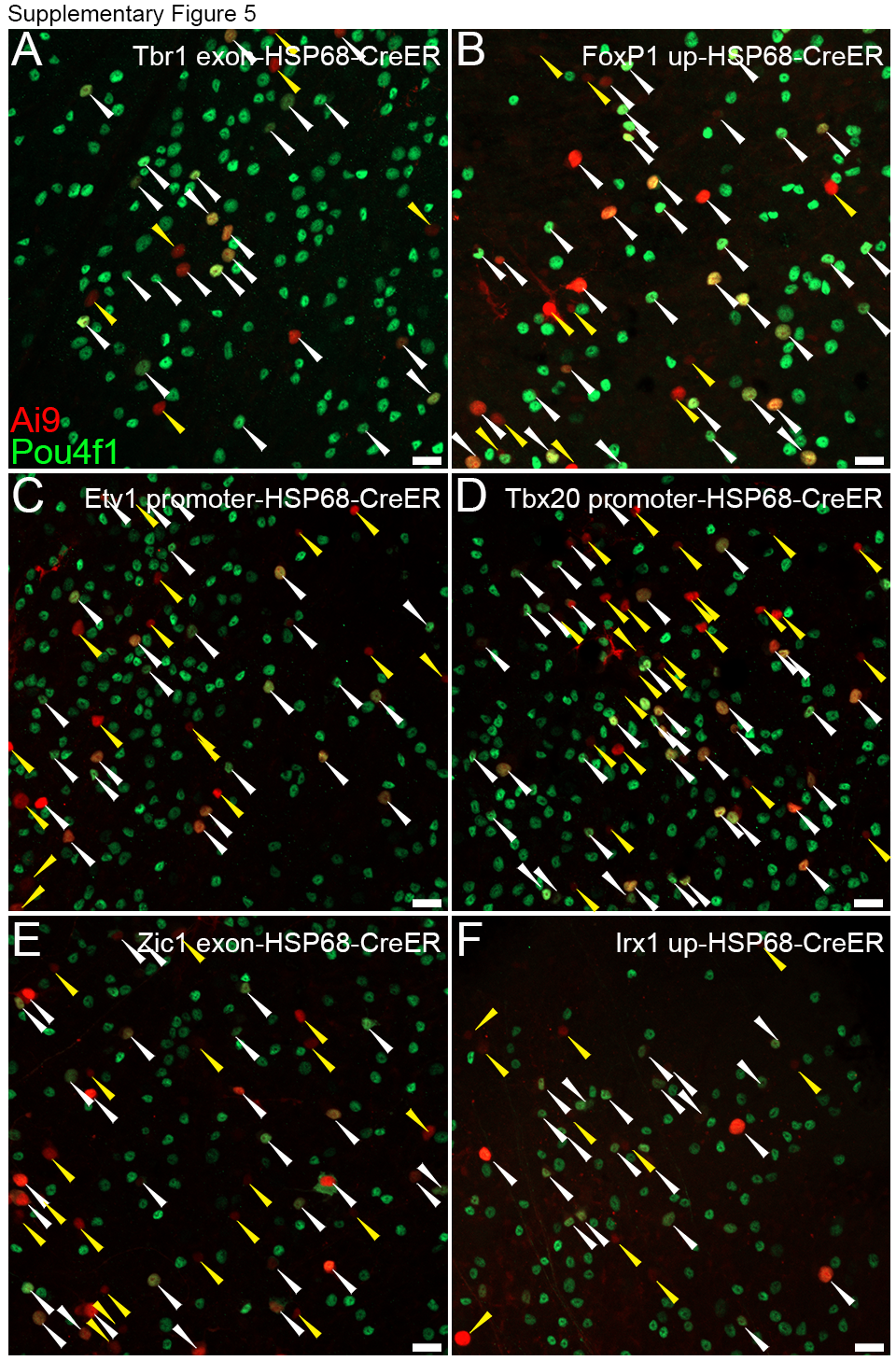

Supplement: Supplementary file 5 [file Image_5.tif]

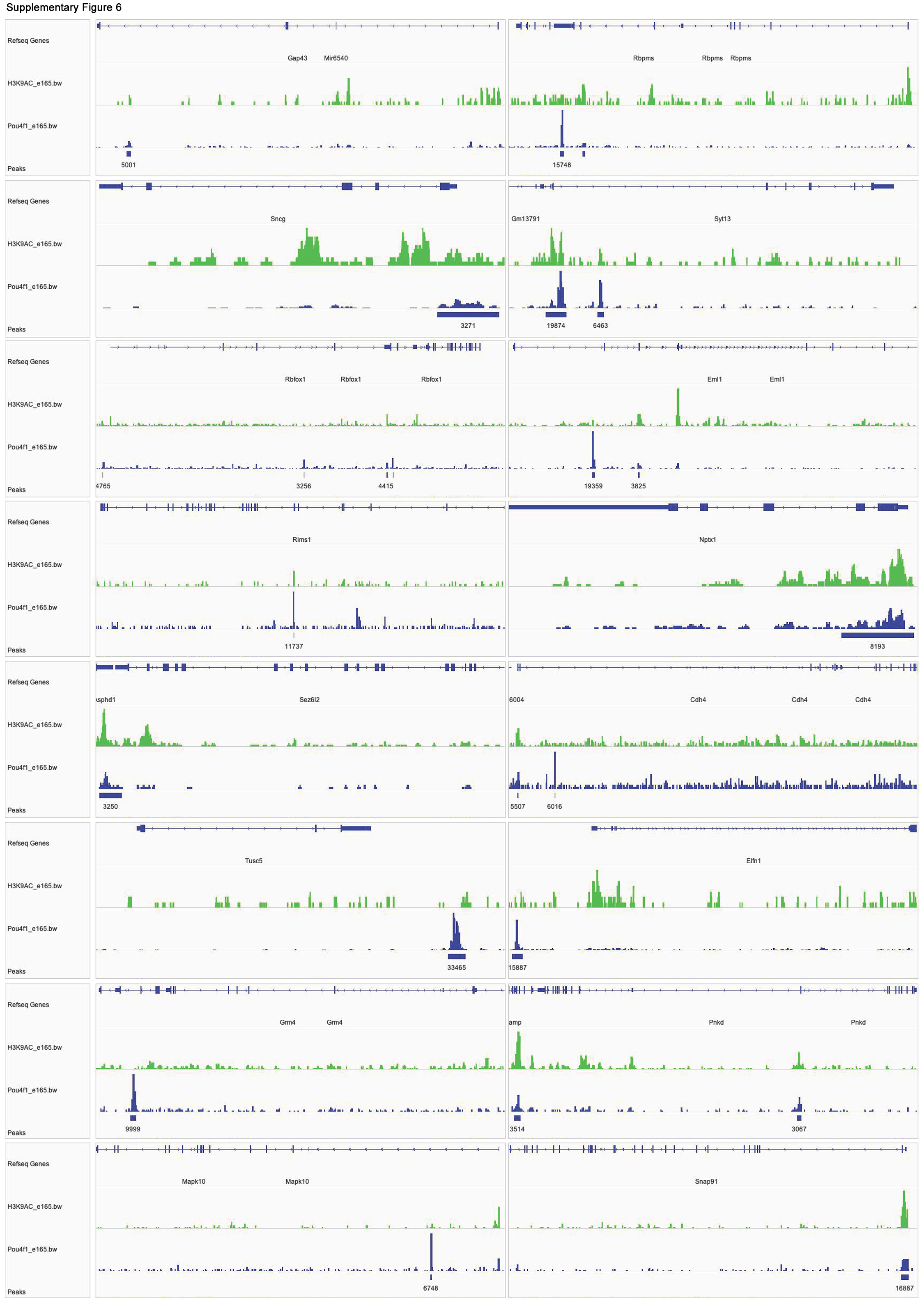

Supplement: Supplementary file 6 [file Image_6.tif]
